# Extracting patient lifestyle characteristics from Dutch clinical text with BERT models

**DOI:** 10.1186/s12911-024-02557-5

**Published:** 2024-06-03

**Authors:** Hielke Muizelaar, Marcel Haas, Koert van Dortmont, Peter van der Putten, Marco Spruit

**Affiliations:** 1 LIACS, Leiden University, P.O. Box 9512, Leiden, 2300RA The Netherlands; 2 Department of Public Health and Primary Care, Leiden University Medical Center, Albinusdreef 2, Leiden, 2333ZA The Netherlands; 3 Department of Business Intelligence, HagaZiekenhuis, Els Borst-Eilersplein 275, Den Haag, 2545AA The Netherlands

**Keywords:** BERT, BERT clinical research, Clinical NLP, NLP clinical lifestyle classification

## Abstract

**Background:**

BERT models have seen widespread use on unstructured text within the clinical domain. However, little to no research has been conducted into classifying unstructured clinical notes on the basis of patient lifestyle indicators, especially in Dutch. This article aims to test the feasibility of deep BERT models on the task of patient lifestyle classification, as well as introducing an experimental framework that is easily reproducible in future research.

**Methods:**

This study makes use of unstructured general patient text data from HagaZiekenhuis, a large hospital in The Netherlands. Over 148 000 notes were provided to us, which were each automatically labelled on the basis of the respective patients’ smoking, alcohol usage and drug usage statuses. In this paper we test feasibility of automatically assigning labels, and justify it using hand-labelled input. Ultimately, we compare macro F1-scores of string matching, SGD and several BERT models on the task of classifying smoking, alcohol and drug usage. We test Dutch BERT models and English models with translated input.

**Results:**

We find that our further pre-trained MedRoBERTa.nl-HAGA model outperformed every other model on smoking (0.93) and drug usage (0.77). Interestingly, our ClinicalBERT model that was merely fine-tuned on translated text performed best on the alcohol task (0.80). In t-SNE visualisations, we show our MedRoBERTa.nl-HAGA model is the best model to differentiate between classes in the embedding space, explaining its superior classification performance.

**Conclusions:**

We suggest MedRoBERTa.nl-HAGA to be used as a baseline in future research on Dutch free text patient lifestyle classification. We furthermore strongly suggest further exploring the application of translation to input text in non-English clinical BERT research, as we only translated a subset of the full set and yet achieved very promising results.

**Supplementary Information:**

The online version contains supplementary material available at 10.1186/s12911-024-02557-5.

## Background

The use of Natural Language Processing (NLP) methods on free (unstructured) text in the clinical domain has risen substantially in recent years. Kormilitzin et al. created Med7, a transferable NLP model specifically tailored to electronic health records [1]. This fine-tuned model performed extraordinarily well on recognising presence of seven categories concerning drug data. Other NLP methods applied to highly specific clinical free text classification tasks, such as classifying axial spondylarthritis and nonvalvular atrial fibrillation, perform similarly well [2, 3].These models are generally trained unsupervised using free text and learn numerical vector representations of words and their context within larger text. 

Over the last few years, Bidirectional Encoder Representations from Transformers (BERT) models have been considered to be state-of-the-art in the natural language processing domain and have seen success within clinical domains, such as with the Med-BERT implementation on electronic health records [4]. These models are generally trained using unsupervised free text and learn numerical vector representations of words and their context within larger text [5]. BERT was the first language model to be able to read text non-sequentially, as well as the first language model that used a transformer-type architecture, which leverages attention mechanisms to process text in parallel and can generate rich contextual word representations [6]. Both these factors contributed massively to BERT becoming the state-of-the-art on a number of common tasks.

Since the creation of BERT, several alterations to its architecture have been proposed with the goal of improving the model’s ability to adapt to a wide variety of tasks. A popular improvement suggestion, the so-called Robustly Optimized BERT pre-training Approach (RoBERTa) model, makes use of a more optimized set of hyperparameters and a more dynamic pre-training task [7]. The application of the RoBERTa architecture has shown to outperform the original BERT model in direct comparisons on a relatively large amount of tasks [7–17]. Over the years, further adjustments have been made to the RoBERTa architecture. One of them in particular, A Lite BERT (ALBERT), makes use of cross-layer parameter sharing and factorised embedding parameterisation as additional inclusions [14]. ALBERT outperforms large versions of both BERT and RoBERTa on the GLUE benchmark, while containing significantly fewer parameters.

As BERT models merely contain the encoder part of a standard transformer architecture, they need to be fine-tuned before they can be used on classification tasks. Furthermore, the BERT architecture can be pre-trained on domain-specific textual data as well in order to obtain a more contextualised version. For example, the ClinicalBERT model was pre-trained on over 2 million English clinical notes and outperforms standard BERT on the task of hospital readmission prediction [18]. Similarly, the BioBERT model that was trained on the same input text as BERT supplemented with PubMed abstracts and full-text articles significantly outperforms BERT on biomedical named entity recognition, question answering and relation extraction [19]. Within the existing literature in the clinical domain, domain-specific models are shown to outperform fine-tuned general BERT models virtually every time a direct comparison takes place, such as for the aforementioned ClinicalBERT [11, 16, 18, 20–22] and BioBERT [11, 19, 21, 23, 24].

In terms of Dutch BERT research, RobBERT has since improved upon the previous state-of-the-art BERTje on sentiment analysis and a disambiguation classification task. In the clinical domain, MedRoBERTa.nl model is pre-trained completely on a collection of Dutch clinical notes [17]. MedRoBERTa.nl is shown to outperform general Dutch language models on a clinical odd-one-out similarity task, as well as on classifying patient mobility levels when fine-tuned. Moreover, the belabBERT model was created, which outperfomed RobBERT on both a sentiment and psychiatric classification task [25].

In current literature on BERT applications, let alone on Dutch clinical BERT text classification, the application of translation of input texts is not very well analysed yet. Nevertheless, it has been shown that for the task of classifying codes from the International Classification of Diseases (ICD) from unstructured German clinical text translating the texts to English resulted in the highest performance [23]. This was most likely the case as translation to English allowed the researchers to be able to use BioBERT, which was trained on English input with a larger size than any of the German models that were included in the paper. Furthermore, BioBERT outperformed standard English BERT on the translated input, further indicating that more domain-specific models are more likely to be suited in comparable situations.

### Problem statement

To date, lifestyle classification in Dutch clinical texts remains largely unexplored. Several investigations focused on the task of extracting smoking status [26–29], while no studies have been conducted into the classification of alcohol use and drug use statuses in Dutch clinical texts. Furthermore, the studies on extracting smoking status use methods which are not considered to be state-of-the-art (anymore) in NLP, such as rule-based string matching, rather than deeper context-aware models such as BERT. As lifestyle classification could aid medical professionals in transforming unstructured text to useful structured fields such as smoking status, there exists a need for insight into the problem-at-hand and possible application of deeper models, as well as a clear research framework for future work into the topic. Furthermore, as stated before, the application of translation to BERT research is severely underanalysed in current literature.

### Aim

In this paper, we assess whether the application of deep BERT models improves upon string matching on the problem of Dutch clinical text lifestyle classification, specifically on smoking, alcohol use and the usage of drugs.

As BERT models are computationally intensive to train and fine-tune, we hypothesise that more shallow, standard classical machine learning approaches, such as models that make use of Stochastic Gradient Descent [30], are a better fit for the problem at hand. For this reason, we compare classical machine learning approaches to BERT and string matching.

We create multiple BERT models and fine-tune these on the task of lifestyle characteristics classification, specifically on smoking, alcohol and drug usage. These BERT models consist of a model pre-trained from scratch, three Dutch BERT models that were further pre-trained on our data and two English models that are finetuned on text that was first translated from Dutch to English. The results of these models are compared to a string matching and classical machine learning approach on a shared test set. Ultimately, between classical machine learning, BERT and string matching we provide one model that performed the best within the context of our experiments and suggest it to be used as a baseline for further research into Dutch clinical text lifestyle classification. The code for our experiments is available on GitHub^1^.

## Methods

In this section we describe our data pipeline, the models we train and how they are evaluated. We furthermore lay out our experiments for identifying a suitable model for the task of lifestyle classification from unstructured Dutch clinical text.

### Data overview

We do not make use of any structured data, such as patient information like age and family history. We furthermore regard every document as the entire context that is available to us for that respective patient. This means that we do not incorporate document history into our data sets, but rather have our data set contain individual, standalone documents which we assume to be unrelated to each other. We obtain full clinical texts to which no alterations have been made before extraction. This means that our classification task concerns a document-level classification, as we wish to assess for each document which lifestyle characteristic statuses apply.

In total we obtained 148 768 unique texts from HagaZiekenhuis, totalling a little over 35 million words. The texts are clinical notes, which consist of consultation notes, anamnesis reports, radiology reports and clinical letters between healthcare professionals. Every text is anonymised using string matching queries. This means patient names, addresses, phone numbers, birth dates, social security numbers and other means for identification are absent from each note. We only extracted texts regarding patients over the age of 18, as this is the minimum age for purchasing alcohol and tobacco in The Netherlands. We also hold the assumption that patients over the age of 18 are more likely to drink alcohol, smoke cigarettes and use recreational drugs.

Table 1 shows the amount of texts and the distribution of the labels for each class. Due to a lack of documentation about previous alcohol and drugs users, we only include “Previous users” for the smoking category. Note that the labelling is done by string matching queries and could differ from a ‘real’ label, as would be annotated by a human. Our string matching queries were constructed in consultation with experts from HagaZiekenhuis and are the product of multiple iterations of data reviewing. The queries can be found in appendix B in the supplementary material.

**Table 1:** Overview and class distribution in query labels from data provided from HagaZiekenhuis

Type of label	Amount of labelled texts	Current users	Previous users	Non-users	No information given
**Smoking**	148 768	7 015 (4.72%)	32 230 (21.66%)	44 677 (30.03%)	64 846 (43.59%)
**Drinking**	143 166	16 017 (11.25%)	-	39 119 (27.32%)	87 940 (61.43%)
**Drugs**	147 999	1 443 (0.98%)	-	53 005 (35.81%)	93 551 (63.21%)

The total amount of labelled texts differs per class, because for one entry (i.e. one patient) the same text can potentially be used for combinations of the smoking, alcohol and drug content column.

During our first experiments with the query-labelled data, we found a near-flawless performance of classical machine learning methods on classifying each lifestyle status. These experiments were done using the classical machine learning methods Random Forest, Stochastic Gradient Descent and Multinominal Naive Bayes. The input for these models consisted of TF-IDF vectorisations of the input text [31]. Experimentation was done on the task of classifying smoking status. For the alcohol and drugs usage classification, in order to adhere to time constraints, we took the 4 best performing models and one lesser performing model and trained them on the alcohol usage and drugs usage classification tasks. The lesser performing model was included as a control group. An example of this can be found in Table 2, in which the performance of these models on the alcohol classification task is shown.

**Table 2:** Alcohol classification task results on test set for four best-performing and one badly performing model

Model name	“No information given” F1-score	“Current user” F1 score	“Non-user” F1 score	Macro F1-score
Stochastic Gradient Descent				
2.1.2 (Ngram 2, Stopwords kept)	1.00	0.99	0.99	0.99
2.1.3 (Ngram 3, Stopwords kept)	1.00	0.99	0.99	0.99
2.2.2 (Ngram 2, Less stopwords)	1.00	0.99	1.00	0.99
2.3.2 (Ngram 2, No stopwords)	1.00	0.97	0.99	0.99
Multinomial Naive Bayes				
1.2.1 (Ngram 1, Less stopwords)	0.95	0.61	0.69	0.75

In Table 2, on all three sub-classes, the Stochastic Gradient Descent models achieve near flawless performance on the alcohol task. In fact, similar scores were achieved on the smoking and drug status classification. This suggests that deeper, more context-aware models like BERT are unnecessary to apply to the problem-at-hand, as these lighter, easier-to-implement shallower models already perform more than acceptably and not much improvement is to be gained. We found, however, that this is not the case. When extracting the most important features and visualising these in word cloud representations, it became apparent the reason for the classical machine learning methods’ high performance was that these models were merely extracting the parameters that were present in the query that labelled the texts, rather than finding any new information i.e. learning new information.

This reliance on query statements poses significant implications for the generalisability and real-world applicability of the classifiers. In scenarios where the query statements do not accurately capture the nuances of label categories the classifier’s performance may severely degrade. As we believe our queries do not wholly capture the extent of possible indicators for the respective classes, this might result in lower performance on unseen texts.

One example of this limitation, for the “non-user” class of the alcohol usage classification task, which represents the patients that are not active alcohol users, important features include “geen alcohol” (no alcohol), “alcohol -”, “alcohol nee” (alcohol no) and “alcohol geen” (alcohol none). All of these features are fully present in our query for labelling patients the non-user class on the alcohol task. Overall, for all three tasks, similar results were found where the most important features were mostly query parameters.

In this light, we conduct an edge case study, where we test the hypothesis that the models did not learn much beyond the query parameters. In order to test this hypothesis, we collect the evaluation score per class for every text. This evaluation score is determined by the model that performed the best on that given task. In order to find edge cases, we extract the top 50 texts that were predicted to be one class with the highest score for another class, as we hold the assumption that these texts have the highest chance of having been misclassified. We then label these texts manually in order to test the accuracy of the query-assigned labels. Ultimately, on these edge case texts an average accuracy of 53% was recorded, which was significantly below the threshold of 90% we set beforehand for acceptable labels. An example of this can be seen in Table 3. As we deem our query labels unsuitable for this reason, we move towards creating hand-labelled input. The query labels are still used however as they serve as our string matching model and are compared to classical machine learning and BERT approaches on the manually annotated data set.

**Table 3:** Alcohol task exempts that were predicted to belong to the non-user class but were misclassified

Edge cases from Dutch clinical notes	Translation	True label
... De patiënt drinkt alcohol (bier), af en toe niet altijd. Opmerkingen mbt alcohol: geen alcohol verslaving. ...	The patient drinks alcohol (beer), occasionally, not always. Notes regarding alcohol:no alcohol addiction.	Current user
Alcoholabusus 2014 SEH-presentatie vanwege alcohol-intoxicatie ... 2020-07: trauma capitis na val van trap bij alcoholintox en speedgebruik. ... Zegt zelf vandaag 3 flessen wijn gedronken te hebben. ...	Alcohol abuse 2014 emergency room appearance due to alcohol intoxication ... 2020-07: trauma capitis after falling down stairs in alcohol intoxication and speed usage. ... Says they drank 3 bottles of wine today...	Current user

These exempts are examples of when the automated labels fail. In the first exempt, the text is labelled as a “Non-user” due to the presence of the word “not” and the phrase “no alcohol”, even though this is erroneous. The second exempt was predicted to be a non-user as well, while it is clear from reading the text that the patient is a current alcohol user. As we found these errors to be too common for us to deem the automatic labels reliable we annotated a smaller subset of the data by hand. The process of which is described in the next section.

### Data annotation

When labelling, every document is regarded as a standalone, complete document about a unique patient, even when multiple texts per patient exist. When a document describes that the patient, for example, will stop smoking at some date in the future (i.e. a date later than the date of the document), it is assumed that the patient is a current smoker. This is a simplification, but it is justified as follows. Firstly, we might not always have the follow-up document about patients, and patient history is inherently incomplete in an evolving patient population. Secondly, in other data sets patients’ notes might not always be easily linked due to privacy conserving regulations, and this procedure makes our current study comparable to such data sets, improving the reproducibility of results.

There are four different kinds of labels that can be assigned to each lifestyle. “**Current users**” are active users of the substance at the time of writing the clinical note, “**Previous users**” have stopped using the substance before the note was written, “**Non-users**” explicitly do not use the substance and the fourth label, “**No information given**”, indicates there is nothing stated in the note about the respective substance. It is important to note that for “Previous users” the time frame of the use does not matter. The label gets assigned regardless of the patient having stopped using the substance a week ago or years ago. The point of reference is the time at which the note was written.

We choose to include the texts we extracted in our edge case study, as we aim to make our resulting models more robust to these edge cases. This collection is comprised of texts that belong to a certain class but are deemed by the highest performing classical machine learning method to have a high chance of belonging to a different class. These edge case texts take up 70% of the texts that were shared among annotators, and around 30% of the total amount of texts. The rest of the texts are chosen from the full data set at random.

There were three human annotators involved in the labelling process. Our annotation guidelines can be found in appendix A in the supplementary material. Besides the first author of this paper (Annotator 1), a university student (Annotator 2) and a healthcare professional (Annotator 3) were involved. After labelling, Annotator 1 served as reviewer, reviewing both annotators’ labels. Wherever discrepancies arose, Annotator 1 and the respective other annotator discussed an appropriate final decision. In total, 1 000 texts were shared amongst the three annotators. Of these texts, the first 500 were labelled by Annotator 1 and Annotator 2, with the remaining 500 getting labelled by Annotator 1 and Annotator 3 respectively. Due to the relatively easy nature of the labelling task and the high agreement, which we touch upon in Tables 4 and 5, the number of annotators and their quality are sufficient. Beyond these 1 000 texts, another 3 700 texts were labelled by hand by Annotator 1 independently, resulting in a total of 4 700 texts that are labelled on smoking, alcohol use and drug use status.

**Table 4:** Cohen’s Kappa values between Annotator 1 and Annotator 2 on the shared data set

	Reviewer	Annotator 1	Annotator 2
**Reviewer**		97.57%	96.81%
**Annotator 1**	97.57%		96.80%
**Annotator 2**	96.81%	96.80%	

**Table 5:** Cohen’s Kappa values between Annotator 1 and Annotator 3 on the shared data set

	Reviewer	Annotator 1	Annotator 3
**Reviewer**		98.09%	97.09%
**Annotator 1**	98.09%		97.09%
**Annotator 3**	97.09%	97.09%	

### Experimental setup

In this paper, we compare string matching, classical machine learning and BERT implementations on our lifestyle classification task. In this section we provide an overview of our experiments. As explained in “Data overview” section, we regard every document as the entire context that is available for a respective patient. For this reason, our classification tasks are performed on a document-level, for as far that is possible adhering to model input size limits.

**Fig. 1:**
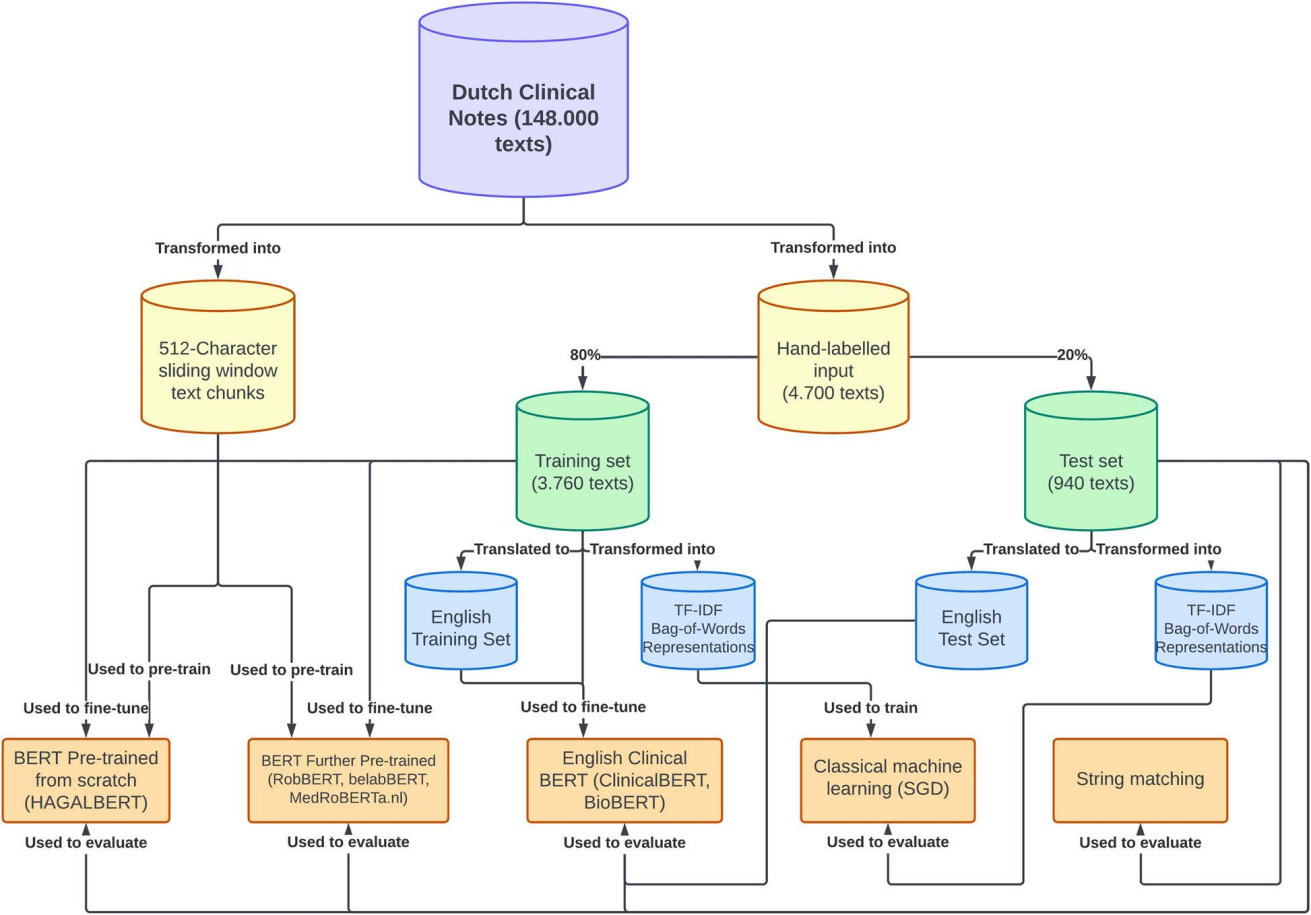
Overview of the experimental setup, showing the workflow on model (pre-)training and evaluation

We compare a string matching approach, a classical machine learning approach in the form of a Stochastic Gradient Descent model and several BERT models on three lifestyle classification tasks, which all classify clinical notes on smoking, alcohol and drug usage status. The BERT models that are compared are either pre-trained from scratch, further pre-trained on top of an existing architecture or fine-tuned on clinical notes that were first translated to English. An overview of our experimental setup can be found in Fig. 1. In this section we elaborate on these processes.

#### String matching

For our string matching approach we utilise the same query we used for automatic labelling, which can be found in appendix B in the supplementary material. For every text in our hand-labelled data set, we acquired its assigned query label and compared it with the manually assigned label, which is the ground truth.

In this paper we make use of a training and test set. Even though a training set is not relevant for string matching, the method does not “learn” anything and therefore does not need a training set, we still only evaluate string matching on our test set to be consistent with the other methods listed below. We elaborate more on our evaluation method and the construction of our training and test sets in “Visualisation” section.

#### Classical machine learning

For our classical machine learning approaches, we use a similar approach to our edge case study described in “Data overview” section. We do not reuse the exact same models, but choose to rerun a random search using the best performing setup. For example, for the alcohol task the Stochastic Gradient Descent model where we set the ngram size to 2 and kept all of the stopwords performed the best, as can be seen in Table 2, so in this experiment we use a Stochastic Gradient Descent classifier with those settings and determine the other parameters via random search. As can be inferred from Table 2, the differences in performance are small. Table 6 shows the best performing setups per lifestyle which we use to train on the hand-labelled data set.

**Table 6:** Best performing setups for classical machine learning for each lifestyle

Lifestyle	Type of classifier	Ngram size	Stopword status
Smoking	Stochastic Gradient Descent	2	Kept
Alcohol	Stochastic Gradient Descent	2	Only negation
Drugs	Stochastic Gradient Descent	2	Kept

#### BERT experiments

We pre-train our BERT model from scratch using the ALBERT architecture. For hyperparameters, we use an optimized subset of architectural parameters for the BERT architecture [32]. For the parameters that do not appear in this subset, we use ALBERT’ standard parameters, as the base ALBERT setup showed to outperform BERT in its respective paper using those parameters [14]. We refer to our from scratch pre-trained model as HAGALBERT, combining the name of HagaZiekenhuis and the name of the ALBERT model architecture. The input that we use to train HAGALBERT consists of the entire bulk of 148 768 texts that were obtained from HagaZiekenhuis.

Our next experiment involves pre-training on top of Dutch BERT models RobBERT, MedRoBERTa.nl and belabBERT. This means we use the models’ pre-trained weights as a starting point and further refine these using unannotated HagaZiekenhuis data. In order to allow for a fair comparison with the first experiment, we set the same hyperparameters as for HAGALBERT. We named the respective models “RobBERT-HAGA”, “MedRoBERTA.nl-HAGA” and “belabBERT-HAGA” and we refer to the models by these names further in this paper.

Our final experiment involves translation. We use *opus-mt-nl-en*, which is a neural translation model, to translate our Dutch clinical documents to English [33]. These types of models are trained on freely available parallel corpora collected in the large bi-text repository OPUS. Opus-mt-nl-en model was tested on the “Tatoeba.nl.en” data set, on which it achieved a chrF-score (CHaRacter-level F-score) of 0.749. This chrF-score is a metric which scores the output of a translation model on the basis of its character n-grams overlapping with the ground truth [34]. It incorporates the character n-gram precision and recall arithmetically averaged over all n-grams. We did not find any other models that were evaluated on this data set, so there exists a possibility that this score is not representative of the quality of the model.

We were unable to translate the full collection of sentence sequences, so only our hand-labelled input was translated. Therefore, pre-training on top of ClinicalBERT and BioBERT was not possible, as we only have 4 700 translated texts, a corpus too small for such training tasks. Instead, these English BERT models were fine-tuned on the translated manually annotated data. Although this could potentially lead to a lower performance, we believe it is interesting to see what the performance of these models is when they are fine-tuned on text that has been translated from Dutch to English. In order to be able to measure the effect of this translation, we also fine-tune ClinicalBERT and BioBERT on the original Dutch hand-labelled text and compare performance directly to the models trained on translated input. An overview of our fine-tuning processes can be found in the next section.

#### Fine-tuning

In the context of BERT research, fine-tuning is done by training a model on tokenized texts and corresponding labels via an appended output layer, in our case a classification layer. We fine-tune all of our BERT models on our hand-labelled data set. Our data set is split into a training and test set, which is uniform among all models. We use the training set to fine-tune our BERT models. We fine-tune every model separately for the smoking, alcohol and drugs labels.

During fine-tuning, we did not make use of hyperparameter optimization. We fine-tune each model for 10 epochs, where we select the model exhibiting the highest performance across these epochs for evaluation on our test set.

#### Evaluation

In this paper, we regard every class within a lifestyle extraction task to be equally important. Furthermore, as there is a class imbalance in both the input data and likely within the real-life context we do not want to reward our model for performing well on a common class but not on uncommon classes. For this reason, rather than using F1-score over all entries in the test set, we calculate the mean F1-score over the classes. This technique is known as Macro F1-score and it disregards the amount of samples per class, making performance on each class equally important [35]. Next to the Macro F1-score, we also record the Macro Precision and Macro Recall, for the same reason. These figures are related to the Macro F1-score and can provide a more detailed view of the performance of our models.

The performance is calculated on our test set, which contains a random 20% portion of the total texts. An overview of the amount of texts per class in the test set can be found in Table 7.

**Table 7:** Class distributions for the smoking, alcohol use and drugs use lifestyle tasks

Set	Non-users	Current users	No information given	Previous users
**Smoking**				
Training	356 (9.47%)	141 (3.75%)	2 532 (67.34%)	730 (19.41%)
Test	84 (8.75%)	38 (4.04%)	611 (65%)	208 (22.13%)
**Alcohol**				
Training	597 (15.88%)	474 (12.61%)	2 673 (71.09%)	16 (0.43%)
Test	147 (15.64%)	142 (15.11%)	648 (68.94%)	3 (0.32%)
**Drugs**				
Training	998 (26.54%)	50 (1.33%)	2 702 (71.86%)	10 (0.27%)
Test	274 (29.15%)	10 (1.06%)	655 (69.68%)	1 (0.11%)

#### Visualisation

Besides Macro Precision, Macro Recall and Macro F1-score we apply visualisation methods on the output of our models in order to explain differences between them. In particular, we use the t-Distributed Stochastic Neighbour Embedding (t-SNE) dimensionality reduction method [36]. We use this method in order to visualize embeddings per class and show how well each BERT model is able to distinguish between the different classes. In this visualisation, we colour every embedding obtained from the text based on its assigned label. Then, under the assumption that the closer the embeddings of the same class are to each other and the further away they are to embeddings of other classes in the visualisation, the better the model is in distinguishing between classes. Using these visualisation methods, we provide insights in the performance of the models, which aspects they perform well on and on which aspects they can improve.

## Results

In this section, we provide performance scores on the lifestyle classification tasks, as well as visualisations that show differences between models.

### Lifestyle classification

For the smoking task, Table 8 shows that the MedRoBERTa.nl model that was further pre-trained on our entire bulk of texts and fine-tuned on hand-labelled texts adhered the best to our requirements, by achieving the highest Macro F1-score. The models that were fine-tuned on translated input perform almost as good as MedRoBERTa.nl, only having a slightly lower Macro F1-score and ClinicalBERT even achieving the highest overall Macro Recall. This is interesting as the translated models were only fine-tuned on the translated collection of hand-labelled texts, rather than further pre-trained on the entire collection of texts as is the case for MedRoBERTa.nl-HAGA, RobBERT-HAGA and belabBERT-HAGA. This indicates that pre-training on a full collection of translated texts could produce even better results. Furthermore, ClinicalBERT and BioBERT performed better when fine-tuned on translated texts rather than the original Dutch texts. This is especially the case for ClinicalBERT, which improves enormously when the input is first translated.

**Table 8:** Results on the common test set for all checked models on the smoking task

Smoking model	Macro precision	Macro recall	Macro F1-score
String Matching	0.93	0.79	0.84
Standard Machine Learning (SGD)	0.83	0.88	0.85
HAGALBERT	0.73	0.68	0.66
RobBERT-HAGA	0.85	0.91	0.87
belabBERT-HAGA	0.61	0.49	0.48
MedRoBERTa.nl-HAGA	0.94	0.93	0.93
BioBERT (original)	0.88	0.87	0.87
BioBERT (translated)	0.90	0.92	0.91
ClinicalBERT (original)	0.42	0.28	0.25
ClinicalBERT (translated)	0.91	0.95	0.92

**Table 9:** Results on the common test set for all checked models on the alcohol task

Alcohol model	Macro precision	Macro recall	Macro F1-score
String Matching	0.74	0.74	0.74
Standard Machine Learning (SGD)	0.71	0.73	0.72
HAGALBERT	0.61	0.54	0.54
RobBERT-HAGA	0.70	0.71	0.71
belabBERT-HAGA	0.64	0.65	0.64
MedRoBERTa.nl-HAGA	0.77	0.80	0.79
BioBERT (original)	0.72	0.73	0.73
BioBERT (translated)	0.72	0.73	0.73
ClinicalBERT (original)	0.73	0.73	0.73
ClinicalBERT (translated)	0.79	0.81	0.80

**Table 10:** Results on the common test set for all checked models on the drugs task

Drugs model	Macro precision	Macro recall	Macro F1-score
String Matching	0.75	0.65	0.68
Standard Machine Learning (SGD)	0.73	0.57	0.60
HAGALBERT	0.46	0.42	0.43
RobBERT-HAGA	0.69	0.60	0.63
belabBERT-HAGA	0.57	0.54	0.57
MedRoBERTa.nl-HAGA	0.86	0.82	0.77
BioBERT (original)	0.50	0.52	0.39
BioBERT (translated)	0.53	0.52	0.52
ClinicalBERT (original)	0.74	0.54	0.57
ClinicalBERT (translated)	0.74	0.57	0.61

For alcohol use, in Table 9, it is clear that our translated ClinicalBERT approach outperforms the Dutch BERT models on Macro F1-score, as well as on Macro Precision and Recall separately. It should be noted that we did not have any query for labelling previous users for string matching. With a query for previous users, the F1-score for string matching would likely be significantly higher. Again, the performance of our translated models is noteworthy as they were not pre-trained on our input data at all, yet ClinicalBERT outperforms the further pre-training and then fine-tuning approach. Interestingly, translation appears to have had little effect on the performance of BioBERT on the alcohol task, while ClinicalBERT, contrastingly, improved. This can be explained by the translated ClinicalBERT model being better able to classify the “Previous user” class on the alcohol task, which the non-translated ClinicalBERT and both BioBERT models are not able to do, resulting in lower Macro performance scores.

For the drugs task, in Table 10, the Dutch MedRoBERTa.nl-HAGA model significantly outperforms the other models. It should again be noted that we did not have a previous user query for string matching for drugs either, which would boost its performance here as well. On this task, the translated models do not perform as well. Again, ClinicalBERT and BioBERT perform better when fine-tuned on translated input than on the original Dutch input.

The lower performance compared to the other tasks could be explained by the skewed distribution among the classes in the drug task. Only 1.3% of the total texts concern current users, while less than 1% of texts concern previous users. This may have lead to the model not having enough data to discern between these classes and the more frequently occurring non-users and “No information given” patients. Furthermore, as the test set only includes 10 current drug users and 1 previous user, the F1-scores on these classes were relatively low, resulting in lower Macro F1-scores.

MedRoBERTa.nl-HAGA was the only model that was able to correctly classify the previous drugs user in the test set, resulting in its Macro F1-score being higher than all of the other models on the drugs task. The F1-scores on the other classes were very similar to translated ClinicalBERT. This indicates that MedRoBERTa.nl-HAGA is more robust to a smaller training set than our translated ClinicalBERT approach.

### t-SNE visualisations

In this section we show t-SNE embeddings for each of our pre-trained models. We only show these visualisations for the smoking lifestyle classification task.

**Fig. 2:**
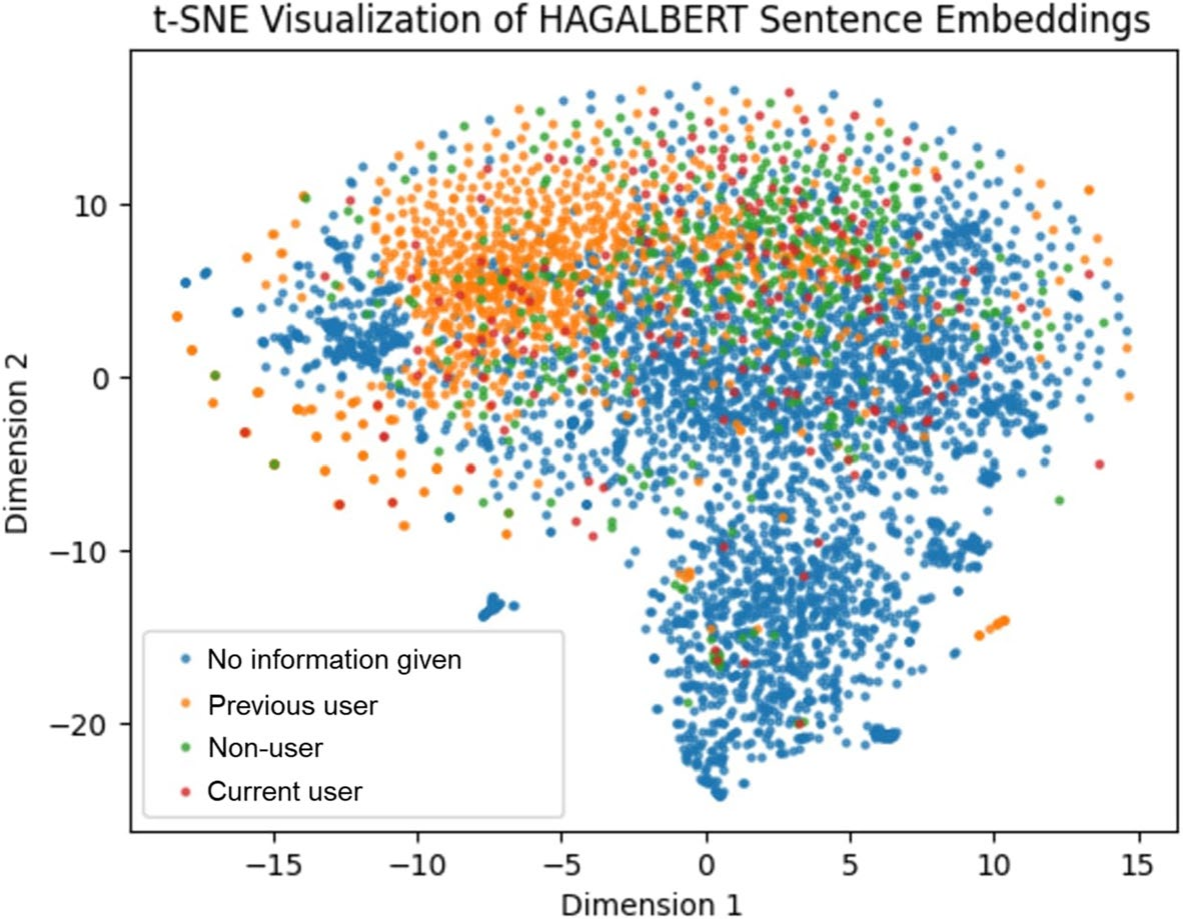
t-SNE visualisation of HAGALBERT embeddings of all hand-labelled texts

**Fig. 3:**
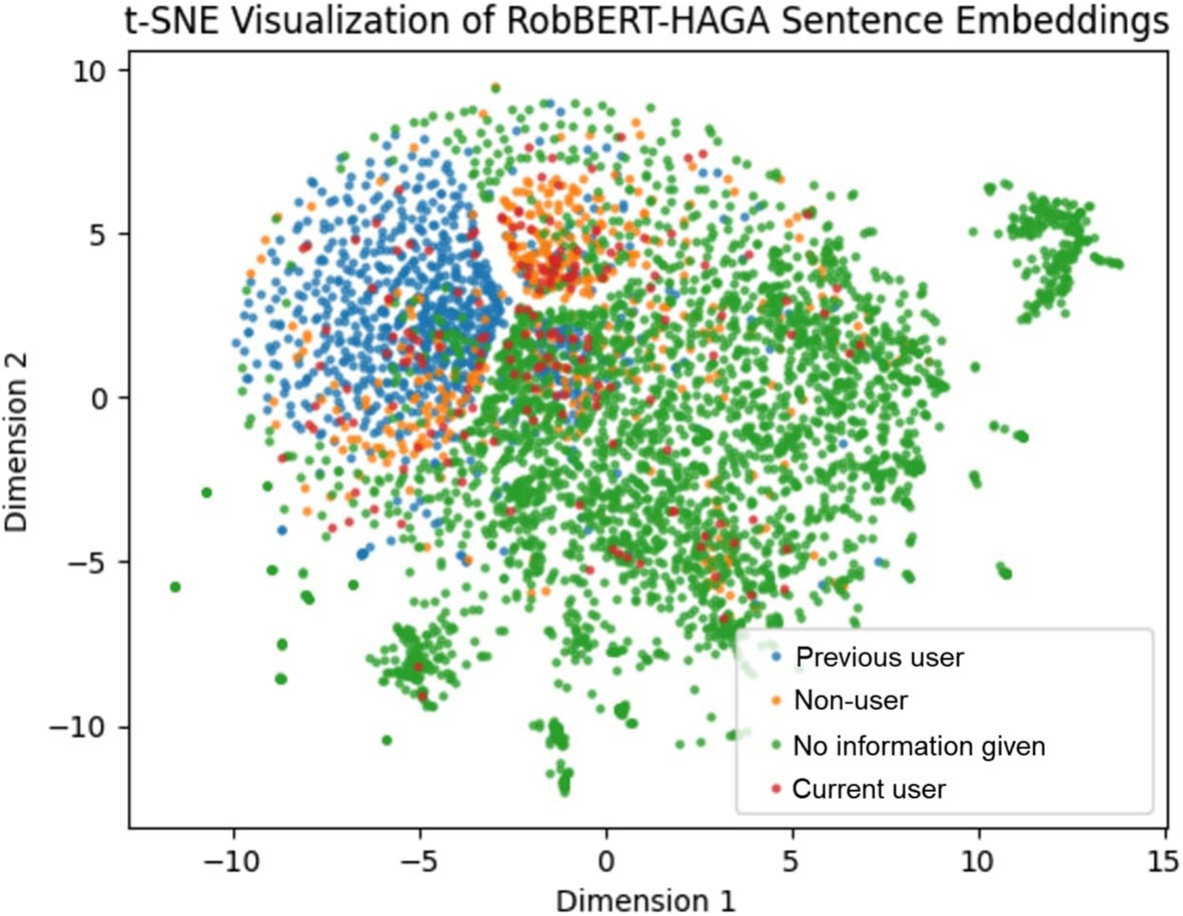
t-SNE visualisation of RobBERT-HAGA embeddings of all hand-labelled texts

**Fig. 4:**
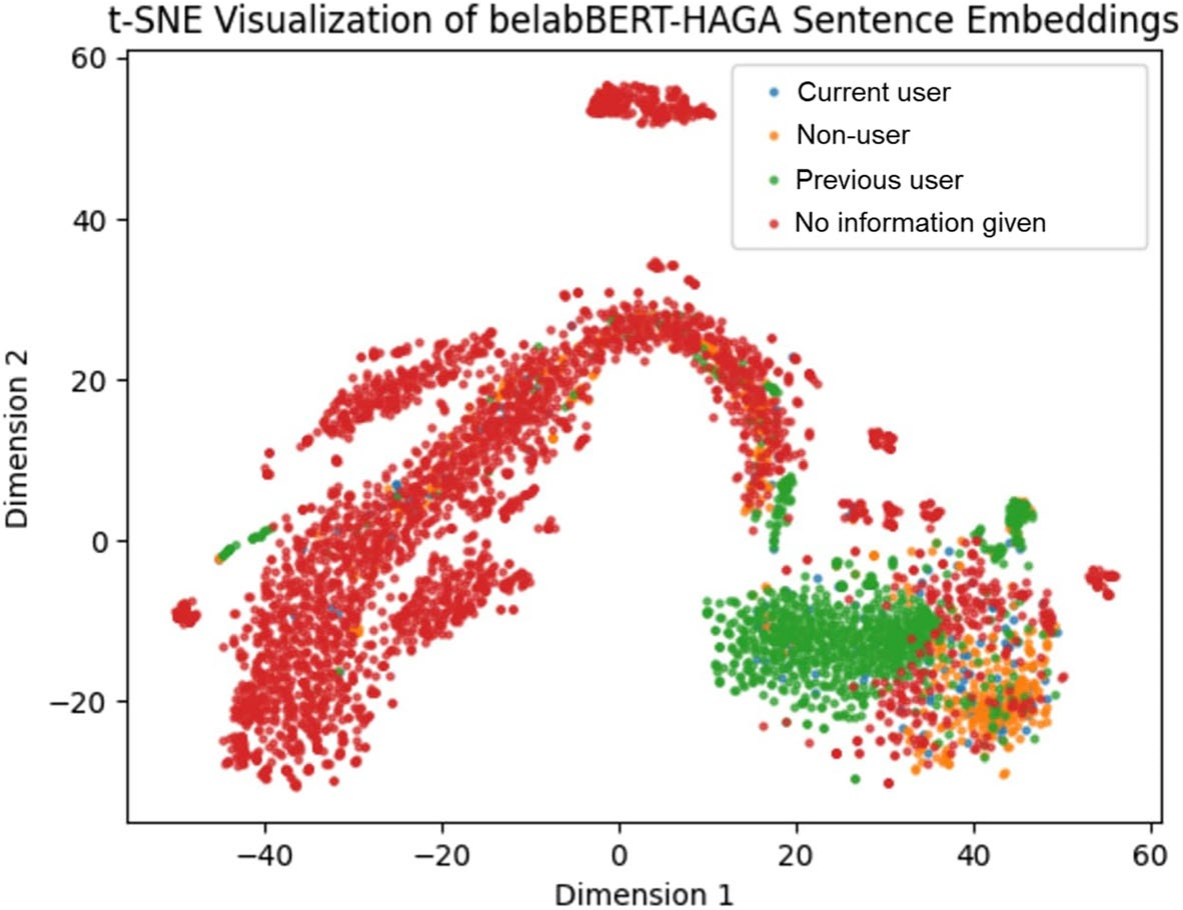
t-SNE visualisation of belabBERT embeddings of all hand-labelled texts

**Fig. 5:**
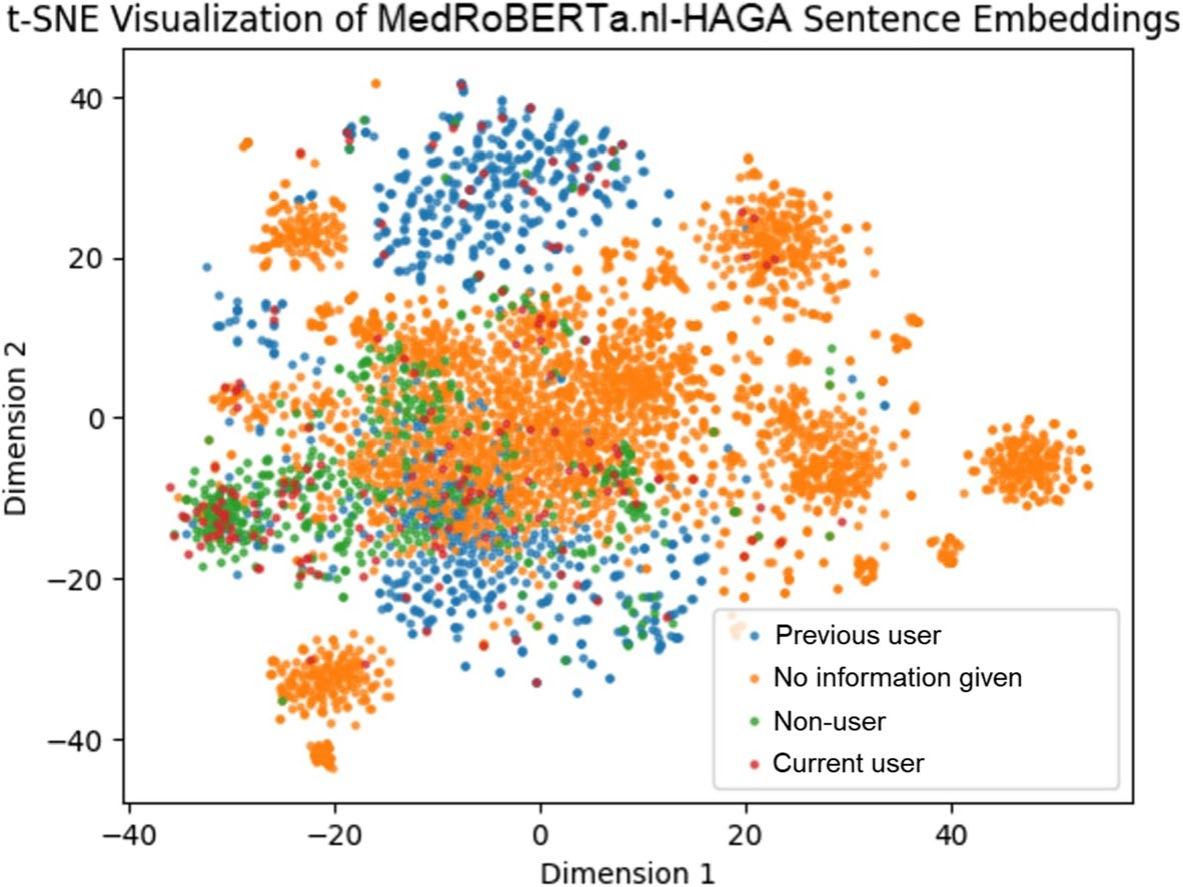
t-SNE visualisation of MedRoBERTa.nl-HAGA embeddings of all hand-labelled texts

For HAGALBERT, Fig. 2 shows that there is a large group of “No information given” texts and a large cluster of “Previous users”. For the other classes, the spread of the embeddings seems random and not many clumps can be identified. This could indicate HAGALBERT is relatively bad at distinguishing between the classes. For RobBERT-HAGA in Fig. 3 this is different, as the distinctions are more clear, and multiple secluded areas with the same label can be identified. Almost all “Previous users” are situated together compared to all over the figure for HAGALBERT, and a large chunk of “No information given” texts can be found. There is also a large clump of “Non-users”. Comparing this figure to HAGALBERT’s, RobBERT-HAGA seems to be more able to group same labelled texts together.

From Fig. 4 we can ascertain that belabBERT-HAGA is worse at distinguishing between the classes than RobBERT-HAGA. It is however adequate at grouping “Previous users” together. “Non-users” appear all over the figure and although some clumps can be identified, their placement still seems too broad to be able to say that the model grasps the classes at all. belabBERT’s F1-score of 0.90 on the “No information given” class was the lowest amongst all models that are visualized in this section and their widely spread distribution in this image compared to the other models explains that. The rest of the classes seem grouped together, but are also common among the “red wave” that can be identified on the image, showing that the model is relatively very poor overall in distinguishing between classes.

Figure 5 clearly shows how MedRoBERTa.nl-HAGA is able to outperform the other pre-trained models on the smoking task. Compared to the other figures, the “No information given” texts are much less spread out, and when they are, they still exist in clumps, showing the model is able to recognise specific types of texts within the “No information given” subset of texts. Furthermore, the “Non-users” also seem much less spread out, showing clumps in various parts of the figure. “Previous users” are weakly co-located, but also occur often in distinct regions of the figure. Interestingly, none of the models seem to be able to group “Current users” together well, indicating that there might be a relatively large amount of ways to recognize current smokers in the document.

## Discussion

We included edge case texts in our manually labelled data set with the goal of having our deeper BERT models adjust to them during fine-tuning. These are texts that belong to one of our classes and are deemed to have a high probability score of belonging to another class, according to the evaluation score of our best performing Stochastic Gradient Descent model. It is unclear exactly how much influence the inclusion of these texts had on the performance of the models. For future research, comparing models that were trained with and without these texts could provide clarification on this regard. Due to limited annotator availability, just 1 000 texts were shared amongst the annotators, while the rest of the texts were solely annotated by the first annotator.

We justify this by showing the inter-rater reliability on these 1 000 texts. This reliability is extraordinarily high. However, because a large portion of the remaining texts were both annotated and reviewed by the same person, bias could be present in this set, as well as possible errors. The chance of this occurring is slim because of the relative easy nature of annotating the texts based on the lifestyle statuses, but it is a risk nonetheless.

Ultimately, the class distribution of the resulting data set was skewed. For each of the three lifestyles, more than two-thirds of the total texts concerned texts that included no information about that particular lifestyle (the “No information given” class). Furthermore, except for smokers, there were almost no previous users for alcohol and drugs present in the data set. This is most likely a product of the actual situation, in that there are overall comparably very few patients that are either previous alcohol users or drug users and/or for which this fact is notable enough for a doctor to note during a consult. It seems that if we were to include more previous users of these substances in the data set that our models would perform way better on these regards, increasing the Macro F1-score.

Our full set of texts, while partly comprised using “edge cases”, might have been not diverse enough due to bias appearing somewhere along the creation process. For example, as the model is trained on query-labelled texts, biases of the query creation process could have influenced the respective model, such as implicit and selection biases. We did not apply any hyperparameter tuning to any of our BERT models to keep within time constraints, which could have hampered performance.

We pre-trained a BERT model from scratch using the ALBERT architecture. This model performed comparably way worse than the models of which we continued the pre-training process with our data. We theorize that this is because of the size of the input set. We had around 148 000 clinical texts in total, with a total size of around 2 gigabytes. In comparison, RobBERT was trained on 39 gigabytes of texts and MedRoBERTa.nl on 13 gigabytes of clinical texts. The results suggest that having more in-domain training data produces higher performance, which could be a large reason for HAGALBERT’s worse performance.

Potentially, using a different BERT model architecture for our pre-training from scratch approach improves its Macro F1-score on the test set, but our hypothesis is that no setup could come close to pre-training on top of other models due to the difference in training size. The ALBERT architecture includes measures like factorized embedding parameterisation and cross-layer parameter sharing [14] and the inclusion of both or either one could also have hampered HAGALBERT’s performance. Our t-SNE visualisations strengthen these points, as HAGALBERT was unable to learn much context compared to MedRoBERTa.nl-HAGA and is worse at distinguishing between classes in the embedding space.

Naively, one might think initially that MedRoBERTa.nl would yield the best results, as it was pre-trained using domain-specific Dutch free text data. As MedRoBERTA.nl-HAGA outperformed the other models on two of the three tasks, it is fair to state that this hypothesis is true within the context of this paper. Interestingly, belabBERT got outperformed by the other models, even though it was trained on more domain-specific Dutch text data than RobBERT. Both of these models are also RoBERTa-based models. Furthermore, in its respective paper, belabBERT outperformed RobBERT in a direct comparison in a sentiment analysis classification task in belabBERT’s source paper [25].

We speculate that the combination of the large amount of input data and the fact that MedRoBERTa.nl is trained on clinical text are the main reasons for MedRoBERTa.nl-HAGA outperforming belabBERT and any other model that was initialized using another model’s weights. For future research on Dutch clinical text lifestyle classification, we suggest either directly comparing MedRoBERTa.nl-HAGA to newer/other models or pre-training on top of MedRoBERTa.nl-HAGA using more input data, in a similar manner as we did to train MedRoBERTa.nl to MedRoBERTa.nl-HAGA.

We translated only the 4 700 hand-labelled texts to English for fine-tuning BioBERT and ClinicalBERT. We did this as we were unable to translate the entire bulk of texts within reasonable time. We used the *opus-mt-nl-en* model for this, because of its low cost and availability within HuggingFace. It could be the case that more complex (neural) translation models like DeepL and Google Translate might improve performance in future research. The translated fine-tuned models performed comparatively really well, outperforming every other model apart from MedRoBERTa.nl-HAGA on the smoking task. ClinicalBERT was even able to outperform MedRoBERTa.nl-HAGA on the alcohol task. Furthermore, we showed that translation of the input to English increases performance for ClinicalBERT and BioBERT, as models fine-tuned on the original Dutch hand-labelled texts performed notably worse.

There are multiple possible explanations for the relatively high performance of English BERT models fine-tuned on translated texts. For one, it could be that pre-training using our bulk of texts curtailed the other models’ classification abilities and that models like RobBERT and MedRoBERTa.nl would perform better on the test set if we had not pre-trained them further but rather fine-tuned them directly like we did for ClinicalBERT and BioBERT. Another reason could again be the difference in domain-specific training data. ClinicalBERT in particular was trained on over 2 million clinical notes, which is comparable to MedRoBERTa.nl, and contains much more clinical data than RobBERT and belabBERT. This could explain why ClinicalBERT performs similarly to MedRoBERTa.nl and outperforms RobBERT and belabBERT on these tasks. Of course this does not take into account that MedRoBERTa.nl was first further pre-trained on our 148 000 clinical notes from HagaZiekenhuis.

The high performance of fine-tuning on translated texts is very promising, and we very strongly recommend to explore this option within other experiments where BERT models are applied. For example, by finding a way to reduce the time needed to translate a text, bigger data sets can be translated within reasonable time and can then serve as input, possibly improving performance even more. We especially recommend taking this approach for Dutch BERT research as, as far as we could find, translating Dutch texts to English and then using English BERT models is a new approach. This approach could also be effective for other languages, and we highly encourage future research into similar approaches on clinical documents in other languages, as the current approach is relatively simple and produces good results for Dutch. A comparative study between models in prevalent and less prevalent languages could help in gaining understanding in how well translation performs relative to the quality of the translation models that exist for that particular language. Performing the same or similar experiments for hospital texts in a different minority language than Dutch could measure the generalisability of our approach, and, if effective, could open a plethora of possibilities and improvements in other languages’ clinical NLP domains. We therefore highly recommend applying our research framework to unstructured clinical texts in other languages.

In terms of clinical relevance, we believe these models could aid medical professionals in improving the registration of lifestyle characteristics in electronic health records. The MedRoBERTa.nl-HAGA model could be used as an advisory system, recommending (per patient) the respective smoking, alcohol usage and drug usage status, based on the most recent clinical notes. Furthermore, the most important features per class could be extracted and used to gain insight into how medical professionals can word these lifestyle characteristics most efficiently for the model to reduce the error rate. Similarly, gaining insight into when the model assigns wrong labels could aid in creating guidelines, guiding medical professionals in writing their notes in a model compliant way, for example. Potential barriers for implementation could be that the model needs to be loaded in from an environment that is compatible with HuggingFace [37], as the model is stored there and makes use of HuggingFace resources. Further problems could arise when the implementing hospital’s text has not been de-identified. As our models are trained on de-identified text, it could be that features from unaltered text influence the models’ performance negatively.

## Conclusion

In this paper, we created and evaluated models for three lifestyle classification tasks on Dutch free text in the clinical domain. For future research, we suggest the MedRoBERTa.nl-HAGA model for further experiments on extracting smoking status and drugs statuses, as this model yielded the highest performance on these tasks. Within the context of this paper we found our best-performing BERT model MedRoBERTa.nl-HAGA to outperform string matching and classical machine learning on our smoking classification task, achieving a Macro F1-score of 0.93, compared to 0.84 of string matching and 0.85 of classical machine learning. On our alcohol classification task MedRoBERTa.nl-HAGA was outperformed however by translated ClinicalBERT, which achieved a Macro F1-score of 0.80, while MedRoBERTa.nl-HAGA achieved 0.79. For the drugs task the same phenomenon occurred as for smoking, with MedRoBERTa.nl-HAGA achieving a Macro F1-score of 0.77, string matching 0.68 and translated ClinicalBERT 0.61.

For future research, we suggest the MedRoBERTa.nl-HAGA model for further experiments on extracting smoking status and drugs statuses, as this model yielded the highest performance on these tasks. Similarly, we suggest translated ClinicalBERT for the alcohol status classification task. In future work, the models from this paper can be further analysed, as well as used as baselines for their respective tasks. We furthermore show the applicability of translation to Dutch BERT tasks, as these models yielded performance that was very close to MedRoBERTa.nl-HAGA, even though these models were only fine-tuned on a small subset of our data. This shows promise for and we advise researchers to apply translation to similar clinical BERT-related experiments in order to explore whether similar or better results can be achieved in comparison with smaller, language-specific BERT models.

### Supplementary Information

Supplementary Material 1.

## Data Availability

No datasets were generated or analysed during the current study.

## Footnotes

1 https://github.com/hielkemuizelaar/clinical-dutch-lifestyle-extraction

## Code availability

Our code is available on GitHub, via https://github.com/hielkemuizelaar/clinical-dutch-lifestyle-extraction.
