# Correction: Extracting patient lifestyle characteristics from Dutch clinical text with BERT models

**DOI:** 10.1186/s12911-024-02575-3

**Published:** 2024-06-17

**Authors:** Hielke Muizelaar, Marcel Haas, Koert van Dortmont, Peter van der Putten, Marco Spruit

**Affiliations:** 1 LIACS, Leiden University, P.O. Box 9512, Leiden, 2300RA The Netherlands; 2 Department of Public Health and Primary Care, Leiden University Medical Center, Albinusdreef 2, Leiden, 2333ZA The Netherlands; 3 Department of Business Intelligence, HagaZiekenhuis, Els Borst-Eilersplein 275, Den Haag, 2545AA The Netherlands

**Correction to: Muizelaar et al. BMC Medical Informatics and Decision Making (2024) 24:151**

10.1186/s12911-024-02557-5.

Following the publication of the Original Article, the authors reported that the Funding statement was incorrect.

**Incorrect**:

This research received no external funding.

**Correct**:

This publication is part of the project “ECOTIP” (with project number NWA.1518.22.151) of the research programme “Dutch Research Agenda Routes by Consortia” (NWA-ORC 2022) which is financed by the Dutch Research Council (NWO).

The original article has been corrected.

