# Supplementary material for: Extracting patient lifestyle characteristics from Dutch clinical text with BERT models
Source: BMC Med Inform Decis Mak. 2024 Jun 3;24:151. doi: 10.1186/s12911-024-02557-5 (PMC11149227; doi:10.1186/s12911-024-02557-5)
Supplement: Supplementary file 1 — Supplementary Material 1. [file 12911_2024_2557_MOESM1_ESM.zip › Supplementary/Appendix A.pdf]

Appendix A: Annotation Guidelines

Extracting Patient Lifestyle Characteristics from Dutch Clinical Text with BERT Models

In this section we indicate for each label which texts do and do not belong to this label. These labels apply to each of the smoking, alcohol usage and drug usage lifestyle characteristics.

When labelling, each text should be viewed as an independent comprehensive entity about a single patient. This means that a text must be approached as if it contains the complete context about one patient and that obtaining more information about this patient outside the text is impossible. This means the text is the complete framework of everything we know about a patient. The text should also be seen as the most recent piece of information available about the patient. For example, if the text states that the patient will quit smoking on May 4, 2019 (the text is from before May 4, 2019), this should be interpreted as a future expression of quitting and the patient should be regarded as a current user, even when the moment of labelling is well after May 4, 2019.

- **Current user**
  A patient is a current user if they are an active user of the lifestyle characteristic in question at the time of writing. The patient is *not* a current user if the text states that they have stopped, but *is* if they intend to stop, for example. Frequency of use does not matter, for example no distinction is made between once a year and once a day. This also means that people who indicate that they only use the respective substance “at parties” or the like should be identified as current users. In cases of doubt, we rely on the suspicion of the doctor in question, if this is mentioned in the text. For example, if the text states that the patient does not answer questions but the doctor indicates that they smell strongly of alcohol, this means that the patient should be regarded as an alcohol user.
- **Previous user**
  A patient is a former user if it is clearly stated in the text that they stopped using the substance in question at the time of writing. If a patient says during conversation that they intend to stop taking the substance, we classify this as a “Current user”. The "Previous user" label therefore only applies to patients for whom it is certain that they have stopped taking the substance and are no longer using it at the time of writing.
- **Non-user**
  A patient is not a user if at the time of writing they are not an active user of the substance in question and the text does not state that they have stopped their usage. If only a negative opinion is given in the text about a substabce (for example, “does not smoke”) and the text does not say anything about the patient's history with the substance, this label should be assigned.
- **No information given**
  If nothing can be found in the text about the respective substance, this label should be assigned. For example, a text could be about an X-ray scan of a patient and no lifestyle characteristics may be mentioned. It also happens that texts contain information about smoking and alcohol but not about drugs, in which case this label must be assigned for drugs. Furthermore, for texts with unclear or conflicting indicators, this label should always be selected. This is the case, for example, when 'smoking +' is mentioned somewhere in a text and further down it says 'smoking -' and this concerns the same patient, without further

elaboration on why this is the case. It also occurs that substances are listed but not filled in, such as: “Smoking: Alcohol: Drugs: “, this also falls under “No information given” for each substance.

Appendix B: String Matching Queries

Extracting Patient Lifestyle Characteristics from Dutch Clinical Text with BERT Models

In this section we provide the queries that were used to label our clinical texts automatically. These queries also serve as our method of string matching, which we compared to our classical machine learning and BERT-like approaches.

Smoking

For our smoking, alcohol and drugs queries multiple conditions were used which were checked in order. In this section we lay out the conditions for every subclass of the smoking lifestyle in this order. Note that if one condition fails the next one is checked and so on. This means that the final class serves as the class to which texts are assigned to when all of the other conditions fail. As the texts are in Dutch, naturally the queries are as well. For this reason, we provide the Dutch keywords within quotation marks and their English translations in parentheses.

1. **Previous user**
   Report contains either:
   - “Rookt niet meer” (Does not smoke anymore)
   - “Gestopt met roken” (Stopped smoking)
2. **Current user**
   Report contains either:
   - “Rookt +” (Smokes +)
   - “Roken +” (Smoking +)
   - “Roker +” (Smoker +)
   - “Rookster +” (Smoker +)
   - “Rookt: ja” (Smokes: yes)
   - “Rookster: ja” (Smoker: yes)
   - “Rookt soms” (Smokes sometimes)
   - “Rookt: soms” (Smokes: sometimes)
3. **Non-user**
   Report contains either:
   - “Rookt -” (Smokes -)
   - “Roken –“ (Smoking -)
   - “Roken: -“ (Smoking: -)
   - “Rookt: -“ (Smokes: -)
   - “Roker –“ (Smoker: -)
   - “Rookster: -“ (Smoker: -)
   - “Rookt niet” (Does not smoke)
   - “Roker: nee” (Smoker: no)
   - “Rookt nooit” (Smokes never)
   - “Rookt: nooit” (Smokes: never)
   - “Roken nee” (Smoking no)
4. **No information given**

Alcohol

For alcohol, the same principles apply as for smoking.

1. **Current user**
   Report contains either:
    - “Alcohol +”
    - “Alcohol: ja” (Alcohol: yes)
    - “Drinkt alcohol” (Drinks alcohol)
    - “Alcohol af en toe” (Alcohol from time to time)
    - “Alcohol per week”
    - “Alcohol week”
    - “Invloed van alcohol” (Influence of alcohol)
2. **Non-user**
   Report contains either:
    - “Alcohol –“
    - “Alcohol: nee” (Alcohol: no)
    - “Drinkt geen alcohol” (Does not drink alcohol)
    - “Geen alcohol” (No alcohol)
    - “Alcohol: geheel niet” (Alcohol: not at all)
    - “Alcohol: geen” (Alcohol: none)
3. **No information given**

Drugs

For drugs, again, the same principles apply.

1. **Current user**
   Report contains either:
    - “Drugs +”
    - “Drugs: ja” (Drugs: yes)
    - “Gebruikt drugs” (Uses drugs)
2. **Non-user**
   Report contains either:
    - “Drugs –“
    - “Drugs: nee” (Drugs: no)
    - “Drugs niet” (Drugs not)
    - “Geen drugs” (No drugs)
3. **No information given**
