# Supplementary material for: Extracting patient lifestyle characteristics from Dutch clinical text with BERT models
Source: BMC Med Inform Decis Mak. 2024 Jun 3;24:151. doi: 10.1186/s12911-024-02557-5 (PMC11149227; doi:10.1186/s12911-024-02557-5)
Supplement: Supplementary file 1 — Supplementary Material 1. [file 12911_2024_2557_MOESM1_ESM.zip › Supplementary/Appendix B.pdf]

Appendix B: String Matching Queries

Extracting Patient Lifestyle Characteristics from Dutch Clinical Text with BERT Models

In this section we provide the queries that were used to label our clinical texts automatically. These queries also serve as our method of string matching, which we compared to our classical machine learning and BERT-like approaches.

Smoking

For our smoking, alcohol and drugs queries multiple conditions were used which were checked in order. In this section we lay out the conditions for every subclass of the smoking lifestyle in this order. Note that if one condition fails the next one is checked and so on. This means that the final class serves as the class to which texts are assigned to when all of the other conditions fail. As the texts are in Dutch, naturally the queries are as well. For this reason, we provide the Dutch keywords within quotation marks and their English translations in parentheses.

1. **Previous user**
   Report contains either:
   - "Rookt niet meer" (Does not smoke anymore)
   - "Gestopt met roken" (Stopped smoking)
2. **Current user**
   Report contains either:
   - "Rookt +" (Smokes +)
   - "Roken +" (Smoking +)
   - "Roker +" (Smoker +)
   - "Rookster +" (Smoker +)
   - "Rookt: ja" (Smokes: yes)
   - "Rookster: ja" (Smoker: yes)
   - "Rookt soms" (Smokes sometimes)
   - "Rookt: soms" (Smokes: sometimes)
3. **Non-user**
   Report contains either:
   - "Rookt -" (Smokes -)
   - "Roken –" (Smoking -)
   - "Roken: -" (Smoking: -)
   - "Rookt: -" (Smokes: -)
   - "Roker –" (Smoker: -)
   - "Rookster: -" (Smoker: -)
   - "Rookt niet" (Does not smoke)
   - "Roker: nee" (Smoker: no)
   - "Rookt nooit" (Smokes never)
   - "Rookt: nooit" (Smokes: never)
   - "Roken nee" (Smoking no)
4. **No information given**

Alcohol

For alcohol, the same principles apply as for smoking.

1. **Current user**
   Report contains either:
   - “Alcohol +”
   - “Alcohol: ja” (Alcohol: yes)
   - “Drinkt alcohol” (Drinks alcohol)
   - “Alcohol af en toe” (Alcohol from time to time)
   - “Alcohol per week”
   - “Alcohol week”
   - “Invloed van alcohol” (Influence of alcohol)
2. **Non-user**
   Report contains either:
   - “Alcohol –“
   - “Alcohol: nee” (Alcohol: no)
   - “Drinkt geen alcohol” (Does not drink alcohol)
   - “Geen alcohol” (No alcohol)
   - “Alcohol: geheel niet” (Alcohol: not at all)
   - “Alcohol: geen” (Alcohol: none)
3. **No information given**

Drugs

For drugs, again, the same principles apply.

1. **Current user**
   Report contains either:
   - “Drugs +”
   - “Drugs: ja” (Drugs: yes)
   - “Gebruikt drugs” (Uses drugs)
2. **Non-user**
   Report contains either:
   - “Drugs –“
   - “Drugs: nee” (Drugs: no)
   - “Drugs niet” (Drugs not)
   - “Geen drugs” (No drugs)
3. **No information given**
